# Effect of Radio Frequency Heating on Yoghurt, I: Technological Applicability, Shelf-Life and Sensorial Quality

**DOI:** 10.3390/foods3020318

**Published:** 2014-05-15

**Authors:** Caroline Siefarth, Thi Bich Thao Tran, Peter Mittermaier, Thomas Pfeiffer, Andrea Buettner

**Affiliations:** 1 Department of Chemistry and Pharmacy, Emil Fischer Centre, Friedrich-Alexander Universität Erlangen-Nürnberg, Schuhstr. 19, Erlangen 91052, Germany; E-Mail: caroline.siefarth@fau.de; 2 Fraunhofer Institute for Process Engineering and Packaging (IVV), Giggenhauser Str. 35, Freising 85354, Germany; E-Mails: thi_bich_thao_tran@cargill.com (T.B.T.T.); peter.mittermaier@ivv.fraunhofer.de (P.M.); thomas.pfeiffer@ivv.fraunhofer.de (T.P.)

**Keywords:** heating, pH, radio frequency, sensory, shelf-life, storage, yoghurt

## Abstract

This first part of a two-part study focuses on the technical feasibility of applying radio frequency (RF) heating at different temperatures (58, 65 and 72 °C) to a stirred yoghurt gel after culturing. For comparison, a convectional (CV) heating process was also applied. The aim was to increase the yoghurt shelf-life, by preventing post-acidification and the growth of yeasts and molds. At the same time, the viability of lactic acid bacteria (LAB) was investigated in view of existing legal regulations for yoghurts. Additionally, the yoghurt color, aroma and taste profiles were evaluated. It was found that the application of RF heating was effective for the rapid attainment of homogenous temperatures of 58 and 65 °C, respectively. For RF heating at 72 °C, it was not possible to establish a stable heating regime, since in some cases, there was significant overheating followed by strong contraction of the yoghurt curd and whey separation. Hence, it was decided not to continue with the RF heating series at 72 °C. In the case of CV heating, heat transfer limitations were observed, and prolonged heating was required. Nevertheless, we showed that yeasts and molds survived neither the RF nor CV heat treatment. LAB were found not to survive the CV treatment, but these beneficial microorganisms were still present in reduced numbers after RF heating to 58 and 65 °C. This important observation is most likely related to the mildness of RF treatment. While post-acidification was not observed on yoghurt storage, slight color changes occurred after heat treatment. The flavor and taste profiles were shown to be similar to the reference product. Furthermore, a trained sensory panel was not able to distinguish between, for example, the reference yoghurt and the RF 65 °C sample by triangular testing (α = 5%), showing the potential of novel strategies for further improvements of heat-treated yoghurt.

## 1. Introduction

Thermal treatment is a common and important strategy in the dairy industry for inactivating microorganisms and enzymes and, thus, guaranteeing safe products throughout the predicted shelf-life. However, traditional thermal treatments rely on heat transfer by conduction and convection, resulting in relatively long heating-up times, depending on the respective food matrix. These limitations can lead to strong physicochemical changes within the product, resulting in sensorial and textural modifications, as well as potentially decreasing the nutritive value. Hence, the dairy industry is always searching for new technologies. Over the last few decades, new technologies have been described in scientific publications, but many have not been broadly transferred to manufacturing processes in the food industry. One of these techniques is the radio frequency (RF) heating of foods with common frequencies of 13.56 MHz and 27.12 MHz. RF heating was first described in the middle of the last century in the context of thawing and curing meat [1,2,3]. The advantage of electromagnetic heating is its ability to generate heat inside the food material by orientation polarization of dipoles, such as water, or the forced movement of ions [4]. In this way, the limitations of conventional heat transfer and heat diffusion are overcome and very rapid heating becomes possible, at least in principle. However, RF heating installations in most cases use very high voltages and are prone to electric flashovers. More recently, RF heating was applied to bottled or packaged food in a water bath equipped with electrodes, as described by Bach [5] and Felke *et al.* [6]. By using water, with its high relative permittivity or so-called relative dielectric constant (ε_r_), instead of air as the dielectric field transfer medium between electrodes and food packages, the electrode voltage can be much reduced without reducing the heating rate. This fact minimizes the risk of electric flashovers. Moreover, the ε_r_ of water is closer to that of food materials than the ε_r_ of air. Exposing food to the electric field in a dielectric environment with similar ε_r_, field concentrations at the edges and corners of packages, which cause local overheating, can be avoided and heating is more uniform. In addition, the preheated water acts as a thermal buffer and provides additional temperature uniformity to the surface of the food package. In order to avoid the absorption of RF energy by the water, de-ionized water is used. 

Microwave heating has also been used to heat milk products, and this has resulted in improved sensorial characteristics compared to conventional heating on a laboratory scale [7]. However, microwave fields with the commonly used frequency of 2.45 GHz have a limited ability to penetrate larger food volumes. The limited penetration is described by the penetration depth parameter. This is the distance from the food surface into the food volume at which 63% of the electromagnetic power has already been absorbed. The penetration depth of microwave fields of 2.45 GHz into milk or yoghurt is in the order of 1 cm, while, according to Felke *et al.* [6], in the case of 27.12 MHz RF fields, the penetration depths into food materials is about 20 cm and, thus, leads to the more uniform heating of products of a larger diameter. A further disadvantage of microwave fields compared to 27.12 MHz RF fields is the presence of patterns of constructive and destructive wave interference inside the food, which leads to patterns of hot and cold spots. In the case of RF fields, the waves are so long, that interference is not relevant on the scale of a food item or a food package.

Due to the demand of consumers for minimally processed, but safe foods, fuelling the need for constant technological progress, heating using radio frequencies was chosen as a promising technological approach for the present study. The aim was to perform a feasibility study on the technological applicability of RF heating on stirred yoghurt gels after culturing. 

Generally, yoghurt is consumed not only because of its high nutritive value and appealing organoleptic properties, but also due to its health-promoting effects. Especially, the living microflora of lactic acid bacteria has a positive influence on human digestion (probiotic effect). Moreover, bioactive peptides that are released, e.g., during fermentation as a result of the enzymatic cleavage of milk proteins, are known to have various effects on human health (biogenic effect) [8]. According to the Code of Federal Regulations [9], yoghurt may be heat-treated to destroy viable microorganisms to extend shelf-life. Nevertheless, if dairy ingredients are heat-treated after culturing, then the name of the food must be followed by the parenthetical phrase “heat treated after culturing”. To increase the shelf-life of yoghurt products beyond three to four weeks at refrigeration temperatures, Kessler [10] describes several temperature ranges that are sufficient for the pasteurization of the yoghurt curds: 65 to 75 °C and holding times of 30 to 60 s. Based on this, a comparable approach was chosen in the present study for post-heating stirred yoghurt filled in glass jars. Three temperature regimes were tested: 58, 65 and 72 °C. In detail, we aimed to prolong the shelf-life by mild heat treatment using an adapted heating technology, reducing the microbial numbers, while, at the same time, maintaining the yoghurt’s sensorial and textural profiles as close as possible to those of the stirred yoghurt reference. RF heating was expected to result in shorter heating-up times compared to convectional (CV) heating and, thus, to impart less damage or deterioration with respect to favorable aspects, such as the sensory properties of the products. A further goal of our study was to determine whether RF heating can achieve the same required homogeneous temperature distribution in the yoghurt curd, as expected from CV heating. Accordingly, for comparison, the yoghurts were also heated via CV treatment using steam as the heat carrier medium. The properties of the treated products (changes in pH, color and sensory properties) were then compared to the stirred yoghurt reference without any additional heat treatment. Additionally, in view of the microbial aspects and the related effects on yoghurt shelf-life, potential changes in microbial numbers (lactic acid bacteria (LAB), yeasts and molds) were investigated. 

A second, separate part of this feasibility study will focus on the changes in microstructure and texture caused by a post-fermentative heat treatment [11].

## 2. Experimental Section

### 2.1. Yoghurt

Commercially available plain stirred yoghurt (4.4% protein, 5.4% carbohydrates, 3.8% fat) was purchased from a local supermarket. The yoghurt was filled in 500 mL glass jars, which were closed with a metal screw cap (twist-off, 70 mm) and were filled up to about 100 mm. The jars had a diameter of 87 mm. All yoghurts were purchased at the same time and had the same best-before date, which was four weeks after purchase. According to the manufacturer disclosure, the yoghurts had a shelf-life of four weeks and, thus, were delivered immediately after manufacturing.

For further investigations, a set-style yoghurt reference (3.8% fat, 3.4% protein, 4.4% carbohydrates), matured in a cup (150 mL), was purchased from a local supermarket and stored with the other yoghurt samples.

### 2.2. Heat Treatment

Post-fermentative heat-treatments were performed within three days after the samples were purchased. Prior to heating, the yoghurt samples were pre-tempered to a controlled starting temperature of 40 ± 1 °C. The following target temperatures were applied: 58, 65 and 72 °C. Immediately after heating, the glass jars were placed in an ice water bath for rapid cooling. Before and after heat treatment (Week 0), the samples were placed into a cooling chamber at refrigeration temperatures of 8 ± 1 °C up to a storage period of five weeks. Thus, the storage period exceeded the yoghurt best-before date by one week. In weekly cycles, various quality parameters were investigated, as explained in the respective sections (Section 2.3, Section 2.4, Section 2.5 and Section 2.6). Samples without any additional heating step were held as references for comparative investigations.

#### 2.2.1. Radio Frequency (RF) Treatment

**Figure 1:**
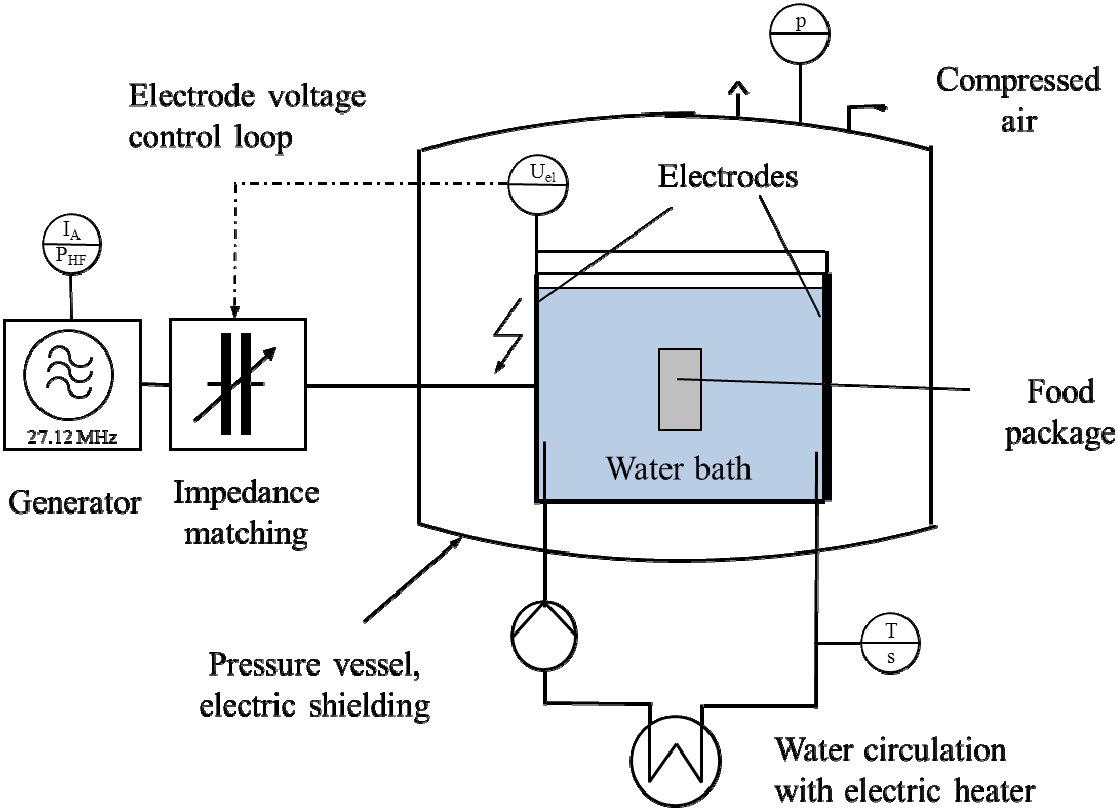
Schematic set-up of the RF water bath, modified from Felke *et al.* [6].

RF heating of yoghurt was performed in an RF water bath on pilot scale, as displayed in Figure 1. RF power was provided by a tube generator with 27.12 MHz operating frequency and 16 kW rated power (Type 16000 K, Kiefel AG, Freilassing, Germany) together with an impedance matching network by the same manufacturer. The power flow to the products was controlled by automatically adjusting the electrode voltage, which was measured directly at the electrodes. Applied electrode voltages during the yoghurt heating trials were between 2.2 and 2.4 kV; with resulting field intensities in the water bath between 17.8 kV·m^−1^ and 19.2 kV·m^−1^. By the additional control of exposure time, the average temperature in the product at the end of RF heating could be determined with a tolerance of ±2 °C. 

**Figure 2:**
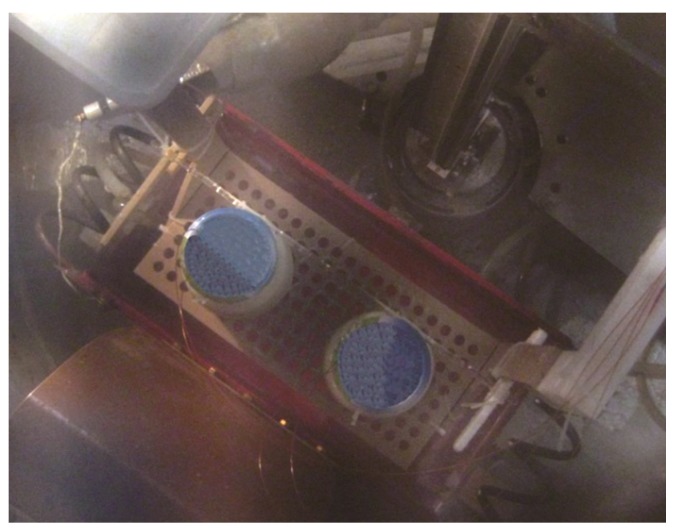
Experimental set-up: Yoghurt jars on a support immersed between the electrodes of the RF water bath (here: inline-temperature measurement with fiber-optic temperature sensors).

Heating procedure: Two glass jars with tightly closed screw caps placed on a support were immersed in the water bath under atmospheric pressure (Figure 2). The water bath was preheated to a temperature slightly above the process target temperature. RF exposure started immediately after immersion and continued for a preset time. The electrode voltages and the exposure times had been determined in previous heating experiments according to the respective target temperatures. Samples for further microbiological, structural, textural and sensory evaluation were exposed to an additional temperature holding time and were not subjected to inline-temperature measurements. However, to guarantee temperature control, three to four spare samples were opened for supervisory purposes during a sample heating series. All details on the specific heating parameters used to produce the product samples are compiled in Table 1.

**Table 1:** Heating procedure of RF heating in an RF water bath.

Process target	Electrode	RF exposure	Holding time ^1^	Water bath
temperature	voltage	time	(RF switched off)	temperature
58 °C	2.3 ± 0.1 kV	60 s	60 s	63 ± 1 °C
65 °C	2.3 ± 0.1 kV	90 s	60 s	70 ± 1 °C
72 °C	2.3 ± 0.1 kV	120 s	60 s	77 ± 2 °C

^1^ At the process target temperature.

Temperature measurements: In previous heating experiments, inline-measurements with fiber-optic temperature sensors (Fotemp 4 fiber-optical thermometer with TS5 0.5-mm diameter sensors, Optocon AG, Dresden, Germany) were performed to adjust the electrode voltage and the exposure times according to the respective target temperatures. The fibers were introduced through septa glued onto holes, which were drilled into the metal screw caps of the jars. Temperatures were measured in the center of the jars, as well as near the walls, at mid-height of the filled yoghurt mass.

During the main series of the heating experiments, manual temperature measurements were performed directly after heating with a fast response thermocouple (0.5 mm diameter, type K, Thermocoax GmbH, Stapelfeld, Germany) connected to an electronic thermometer (Ebro TFN 520-SMP, Ebro GmbH, Ingolstadt, Germany). Temperatures were measured at different height levels within the yoghurt mass in the jars. In addition, the average temperature of the yoghurt filling was measured after mixing with a plastic spoon, because of its low thermal capacity.

The electric conductivity (mS·cm^−1^) of the stirred reference yoghurt was measured with a laboratory conductometer (Innolab Cond level 2, WTW GmbH, Weilheim, Germany) within a temperature range of 25 to 55 °C.

#### 2.2.2. Convectional (CV) Treatment

CV heating of yoghurts was performed in a convection oven (Rational white efficiency SCC WE61, Rational AG, Landsberg am Lech, Germany) in steam mode with 100% saturated steam. 

Heating procedure: Five glass jars with tightly closed screw caps were placed on a fence that was positioned in the center of the convection oven. The oven temperature was set to slightly above the process target temperatures. An inline-thermocouple was used to control the products temperature, with its tip placed in the core of one additional yoghurt jar. Therefore, the lid of the jar was perforated to yield a center hole; this temperature control sample was discarded afterwards. The samples were exposed to CV heating until the process target temperature was reached and an additional temperature holding time was maintained. All details on the respective CV heating parameters used to produce the product samples are compiled in Table 2.

Temperature measurements: In addition to the inline-thermocouple, the entire temperature profile was recorded once by the use of a fast response thermocouple (0.5 mm diameter, type K, Thermocoax GmbH, Stapelfeld, Germany), placed in the core of the product and connected to a data logging instrument (ALMEMO 2890-9, Ahlborn Mess- und Regelungstechnik GmbH, Holzkirchen, Germany).

**Table 2:** Heating procedure of convectional (CV) heating in a convection oven (100% saturated steam).

Process target temperature	CV exposure time	Holding time ^1^	Oven/ steam Temperature
58 °C	60.0 ± 0.5 min	60 s	63 °C
65 °C	61.5 ± 4.5 min	60 s	70 °C
72 °C	58.0 ± 0.5 min	60 s	77 °C

^1^ At process target temperature.

### 2.3. Microbiological Investigations

The microbial numbers of yeasts and molds, as well as lactic acid bacteria (LAB) were investigated directly after (post-)manufacturing of the different yoghurt samples (Week 0) and after a storage period of five weeks. No further inoculation of the samples with LAB or other microorganisms was performed, and the jars were opened just immediately before the samples were taken for further microbial investigations. For the preparation of sample dilutions, 10 g of yoghurt were dissolved in 90 mL of Ringer’s solution (25%, OXOID LTD., Hampshire, U.K.). Ringer’s solution was also used for any further dilution steps. In detail, for each treatment and temperature regime, three independent yoghurt jars were randomly selected and three dilution steps were prepared in threefold repetition to analyze microbial numbers. 

Yeasts and molds: yeast extract glucose chloramphenicol (YGC) agar (Merck KGaA) was selected as the medium for the viability investigation. The incubation was performed at 25 °C aerobically for three to five days. LAB: de Man, Rogosa and Sharpe (MRS) agar (Merck KGaA) was used for enumeration, and aerobic incubation was performed at 37 °C for 72 h. Microbial numbers were expressed as colony forming units (CFU) g^−1^.

### 2.4. pH and Color Measurement

The determination of the pH of the yoghurt samples was performed over the entire storage period (Weeks 0, 2, 4 and 5) using a pH meter (pH 538, WTW GmbH, Weilheim, Germany) together with a pH electrode (BlueLine 11pH, SI Analytics GmbH, Mainz, Germany) and temperature electrode (TFK 325, WTW GmbH). Instrumental color analysis was carried out directly after manufacturing (Week 0) by the use of a Chroma-meter CR-300 (Konika Minolta Inc., Marunouchi, Japan) with a DP-301 data processor. Calibration was performed on a white standard (CR-A43, Konika Minolta Inc.). The stirred samples were filled in Petri dishes, and the surface was sleeked. Each sample was analyzed at ten different points above the surface, and the color was expressed in L*a*b* mode, in which L* represents the lightness value and a* and b* values the chromaticity coordinates. For color and pH measurements, the samples were analyzed in triplicate.

### 2.5. Aroma and Taste Profile Analysis

Stirred yoghurt samples (20 mL) were filled into sensory glass beakers (140 mL, J. Weck GmbH u. Co. KG, Wehr, Germany) and closed with a lid. Sensory analyses were performed in a sensory panel room at 21 ± 1°C. Trained panelists (*n* = 12, male/female, age 24 to 45) with normal olfactory and gustatory function participated in the sensory sessions and exhibited no known illness at the time of examination. Prior to this study, the assessors were recruited in weekly training sessions in the recognition of about 100 selected odor-active compounds according to their odor qualities by means of an in-house developed flavor language. The order of the presentation of the different yoghurts was randomized, and no information on the purpose of the experiment or the composition of the samples was given to the panelists. The results were averaged for each attribute. Sensory analyses were performed up to Week 4 of storage (best-before date) in intervals of two weeks (Weeks 0, 2 and 4). 

Aroma profile analysis (APA): in the first session, the panelists were asked to evaluate the yoghurt gels (retronasal), and the named odor attributes of the different products were collected. Attributes that were detected by more than 50% of the panelists were selected for subsequent evaluations. In subsequent sessions, the panelists were asked to score the perceived retronasal intensities of the selected attributes on a seven-point-scale from 0 (*no perception*) to 3 (*strong perception*) in increments of 0.5. 

Taste Profile Analysis: in addition to the APA, the panelists were requested to evaluate the following taste attributes: sweet, sour, salty and bitter. The panelists had to score the attributes’ intensities on a visual analogue scale from 0 (*not perceivable*) to 10 (*strongly perceivable*).

### 2.6. Triangle Test

A triangle test was performed according to DIN EN ISO 4120:2007 [12]. Two triangles of the following yoghurt gels were tested: reference *vs*. RF 65 °C and RF 58 °C *vs*. CV 58 °C. During the tests, the panelists had their eyes bandaged to avoid any influence of the possible differences in the yoghurts’ appearance. Twelve panelists evaluated each triad in duplicate, leading to 24 evaluations in total. The test was performed after a storage period of four weeks (best-before date).

### 2.7. Statistical Analysis

Statistical analyses were performed by the use of the software OriginPro 9G (OriginLab Co., Northampton, MA, USA) and Statistica 10 (StatSoft Europe GmbH, Hamburg, Germany), respectively. For all groups of data, one-way analysis of variances (ANOVA) and Fisher LSD post-hoc testing were carried out to elaborate differences between the differently treated yoghurts and during storage (repeated measures one-way ANOVA). The level of statistical significance was set at 5%.

## 3. Results and Discussion

This section presents the results of the RF and CV heating of yoghurt products after culturing and describes the technological applicability, shelf-life, pH, color and sensorial changes. The details of the textural and microstructural changes are given elsewhere in the second part of this two-part study [11].

### 3.1. Technological Applicability of the RF Heating of Yoghurt Gels

**Figure 3:**
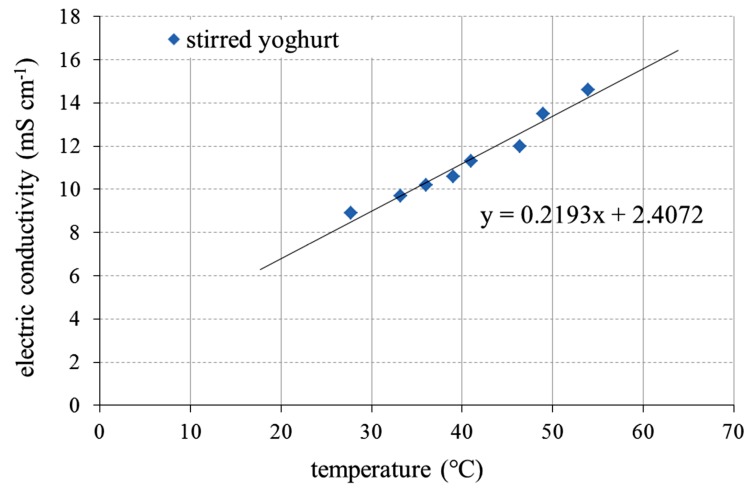
Electric conductivity of the plain stirred reference yoghurt.

To get initial information about the electrical properties of the plain stirred reference yoghurt, its electric conductivity was measured. As can be seen in Figure 3, the electric conductivity of the yoghurt was directly proportional to the temperature. Generally, the measured electric conductivity was slightly higher than the literature values for milk, most likely due to the contribution of lactic acid to conductivity [11].

**Figure 4:**
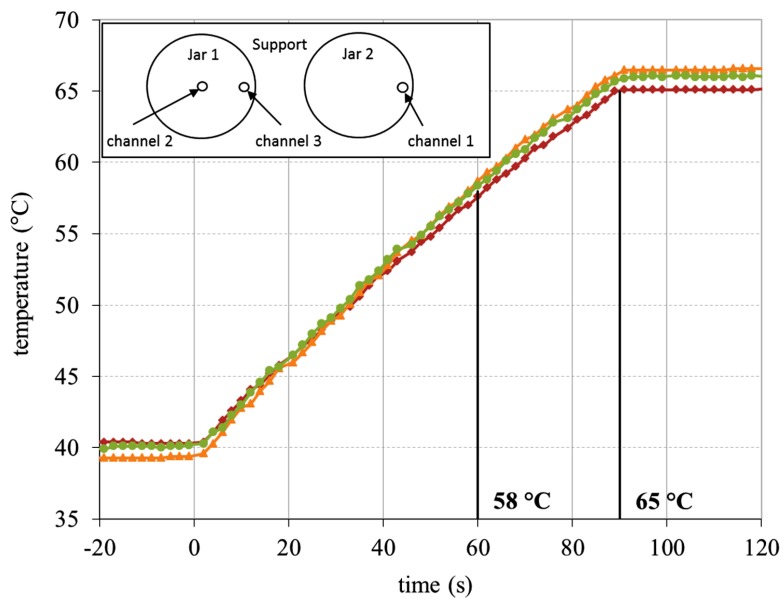
RF heat treatment: the temperature-time profile for heating a stirred yoghurt (in glass jars) to 65 °C in an RF water bath (T = 70 °C).

RF heating was carried out as described in Section 2.2.1, and three different temperature regimes were applied, which were chosen based on the death line of vegetative cells [10]. Starting at a temperature of 40 °C, 60 s were necessary to reach 58 °C, 90 s to obtain 65 °C and 120 s to achieve 72 °C. Thus, the heating rate for all temperature regimes was 0.28 ± 0.02 K·s^−1^ (*cf*. Figure 4.)

Besides a fast heating rate, temperatures of 58 and 65 °C could also be applied very homogeneously to stirred yoghurt in an RF water bath. After heating yoghurt jars to 72 °C, in some cases, there was significant overheating followed by the strong contraction of the yoghurt curd and whey separation. It was not possible to establish a stable heating regime for a 72 °C process. The reason for the heating instability is not clear. A possible explanation could be a sudden change in the dielectric properties above 70 °C, which provoked faster heating. It was therefore decided not to undertake an RF heating series at 72 °C, and this is the reason why no temperature-time profile at 72 °C is shown in Figure 4. Further details concerning syneresis and the texture analysis of the few yogurt samples that were heated to 72 °C are described in the second part of this two-part study [11].

In the frame of this feasibility study, experiments were performed on a small pilot scale. However, RF technology with power ratings of up to several 100 kW are currently implemented on the production scale for manufacturing processes in general industries, including the food industry. Furthermore, large pilot scale equipment of the RF water bath heating process used in this study exists, and its economic feasibility has been estimated [13]. Although investment costs are higher for the RF heater compared to conventional technologies, the energy costs are comparable, even when taking into account the electricity usage compared to the fossil fuel consumption of conventional heaters. Notably, energy costs with emerging RF power generators constructed with semi-conductor technology are expected to fall, due to their higher efficiency, and they are more robust than traditional tube generators.

### 3.2. Technological Applicability of CV Heating of Yoghurt Gels

**Figure 5:**
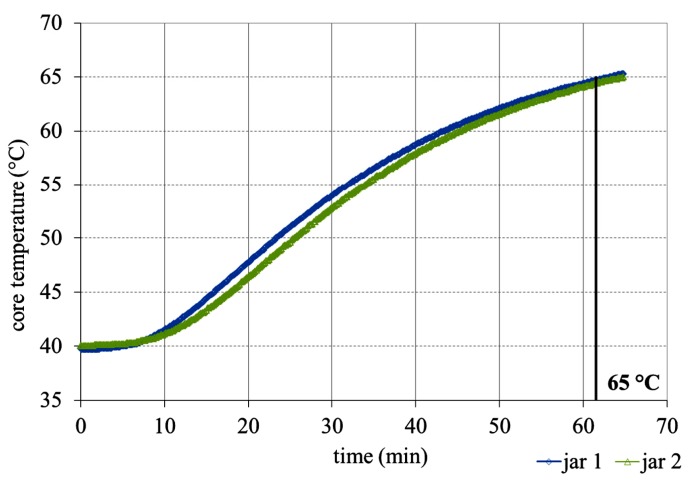
CV heat treatment: temperature-time profile for heating a stirred yoghurt (in glass jars) to 65 °C in a convection oven in steam mode (100%, T = 70 °C).

Temperatures of 58, 65 and 72 °C were also applied to stirred yoghurt in a convection oven. Unlike with RF heating, heat transfer limitations were encountered in the convection oven. This was even so when the heat energy was transferred using steam as the heat carrier medium rather than air. The result was that the heating rate was comparatively low, with the heat curve showing a slowly ascending sigmoidal behavior. Unlike with RF heating, different heating rates for each temperature regime were observed: 0.30, 0.41 and 0.55 K·min^−1^ (58, 65 and 72 °C), respectively (Figure 5).

While difficulties were reported for the dielectric heating of yoghurt gels to 72 °C, CV heating was successfully applied to all temperature regimes. However, the aim of the present study was to improve yoghurt shelf-life, while maintaining its high quality with regard to living LAB and the expected texture and sensorial properties. As significant textural changes occurred in the CV 72 °C samples, as reported in the second part of this study [11], these products were produced in the same small batch numbers as the RF 72 °C samples.

### 3.3. Microbiological Investigations

Bach [5] was the first and, to the best of our knowledge, the only scientist who has applied mild temperatures to yoghurts filled in plastic containers via electromagnetic fields. Nevertheless, no detailed information was provided about microbial numbers.

**Figure 6:**
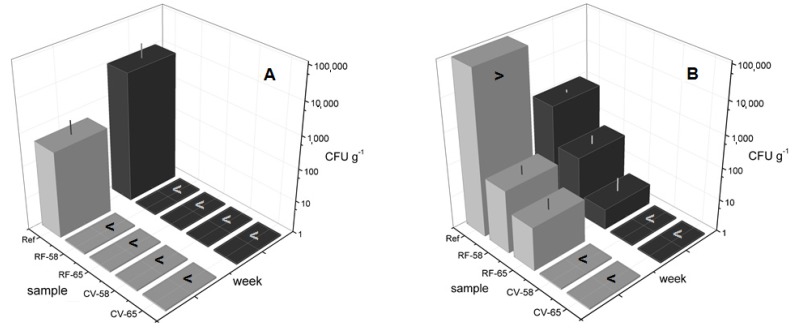
Colony forming units (CFU) g^−1^ at Week 0 (grey) and Week 5 (black): (**A**) yeasts and molds, (**B**) lactic acid bacteria (LAB). CFU g^−1^ are expressed as mean values ± standard deviation (SD), with “>” denoting “more than” or “<” denoting “less than” the proposed CFU g^−1^.

Thus, LAB, as well as yeasts and molds were characterized in the heat-treated yoghurt products of the present study. Microbiological investigations were performed directly after manufacture and also after a storage period of five weeks. The results are shown in Figure 6.

In Figure 6, the reference yoghurt without any additional heat treatment (Ref) demonstrates the initial population (Week 0) of yeasts and molds (A) and LAB (B). Generally, a reduction in microbial numbers was observed after any additional heat treatment of the yoghurt (Figure 6). In detail, yeasts and molds did not survive post-fermentative RF and CV heat treatments, even at 58 °C. On the other hand, LAB were found to partially survive RF heating to 58 and 65 °C, while they were inactivated by CV heating at the same temperatures. The decrease in LAB numbers was in good agreement with the temperature and time of heat treatment (thermal stress).

Regarding RF and microwave processing, there is an ongoing discussion about the possible non-thermal effects on microorganisms due to exposure to an electrical alternating field. It was shown by Heddleson and Doores [14] that the inactivation of microorganisms can be attributed to the heat energy developed within the product, which was also described by Datta and Davidson [4] on comparing the inactivation curves of microorganisms from RF, microwave and conventional heating. These observations are further supported by our data on LAB numbers, which are in good agreement with the degree of thermal stress applied to the yoghurts. Accordingly, our work shows that RF is a mild heating method.

Generally, these results are in agreement with the literature findings on the thermal lability of these microorganisms (*cf*. Section 1). Kessler [10] described that mild heat treatment at 50 to 55 °C for several minutes might be sufficient for fermented milk products in view of the targeted destruction of yeasts, molds and rods that produce much acid and whose proteases are able to break down proteins, yielding unwanted bitter peptides. It might be that even milder temperatures suffice to prolong yoghurt shelf-life. However, this should be investigated in more detail in a further study.

Within the framework of this feasibility study, microbial numbers were also investigated after five weeks of storage. In the case of the stirred reference yoghurt, an increase in the number of yeasts and molds was found in storage (Figure 6A), while LAB numbers were generally found to decrease in storage (Figure 6B); an observation that is in accordance with previous reports on LAB numbers [15,16]. Summarizing our results, one can conclude that the RF heating of yoghurt showed promise: an increased shelf-life can be predicted, since a reduction in microbial numbers was achieved. Hence, the potential to prolong yoghurt shelf-life is related not only to the inactivation of yeasts and molds, but also to the prevention of post-acidification due to reduced LAB numbers. This assumption follows from the work of Chandan and O’Rell [17], who described that heating to 60–65 °C stabilizes the product, so that the yoghurt shelf-life is prolonged to eight to 12 weeks (after manufacture) at a storage temperature of 12 °C or lower. In a follow-up study, the experimental design should therefore include a storage time up to at least three months. 

### 3.4. pH and Color Measurement

The pH value was measured over the entire storage period in order to reveal possible post-acidification due to the activity of living LAB. A storage period of up to five weeks did not reveal any changes in pH. The pH stayed very constant at a value of 4.3 ± 0.1 (10.8 ± 0.6 °C) for all samples. Thus, any ongoing post-acidification can be excluded, also for the reference yoghurt, without any further heat treatment and, thus, high LAB numbers. 

Additionally, color changes in the yoghurt gels due to post-fermentative heat treatment were investigated directly after manufacture (Table 3). It was found that the color between the different yoghurt matrices differed slightly. The L* value (whiteness), negative a* value (green fluorescence) and positive b* value (yellow fluorescence) significantly increased after an additional heat treatment at 58 and 65°C. A direct correlation to the final temperature applied was shown for the positive b* value and, thereby, a change towards a yellow color. Typically, yoghurt shows a blue fluorescence. This fluorescence occurs due to the conversion of riboflavin (vitamin B2), which has a characteristic yellow to green fluorescence, to colorless leucoriboflavin in the presence of LAB [18]. This explains the lower b* value of the reference yoghurt. An increase in the yellow character of the yoghurts with the increasing temperature of the heat treatment might be due to a slight caramelization processes; Maillard reactions are less probable, due to the low pH of yoghurt and the mild temperatures applied. Another reason might be the conversion of riboflavin to lumiflavin, which also shows a yellow-to-green fluorescence, but again, such reactions are favored in alkaline solutions [18].

To the best of our knowledge, no other literature describes the color changes in yoghurt due to an additional heating step after culturing.

**Table 3:** Color changes in yoghurt due to post-fermentative heat treatment and the results of ANOVA (one-way).

Color analysis (L*a*b*)	Reference yoghurt	RF-treated yoghurts			CV-treated yoghurts			ANOVA results	
		58 °C	65 °C	72 °C	58 °C	65 °C	72 °C	*F* value	*p* value
L*	90.36 ± 0.08 b	90.71 ± 0.05 d	90.63 ± 0.02 c,d	89.51 ± 0.10 a	90.67 ± 0.04 c,d	90.59 ± 0.09 c	90.45 ± 0.04 b	119.27	3.33 × 10^−11^
a*	−3.34 ± 0.06 a	−3.47 ± 0.04 b,c	−3.45 ± 0.04 b,c	−3.73 ± 0.02 d	−3.49 ± 0.01 c	−3.48 ± 0.01 c	−3.42 ± 0.03 b	37.99	7.05 × 10^−8^
b*	9.75 ± 0.17a	10.31 ± 0.05 b	10.27 ± 0.06 b	11.12 ± 0.12 d	10.41 ± 0.10 b	10.33 ± 0.09 b	10.66 ± 0.10 c	47.11	1.72 × 10^−8^

Intensity values with different letters indicate significant differences between products (*p* < 0.05, Fisher LSD post-hoc).

### 3.5. Aroma and Taste Profile Analysis

The aroma profile of yoghurt is a mixture of various odor-active compounds [19,20,21]. Further characteristics of yoghurt flavor are, for example, the sour impressions and the specific textural properties that are jointly evaluated when tasting yoghurt [22,23]. In the present study, the heat-treated yoghurt samples and the stirred reference yoghurt were evaluated retronasally by means of an APA and taste profile analysis in order to reveal possible profile changes due to an additional heating step (Table 4).

Regarding the APA, the following descriptors were detected in the yoghurts by more than 50% of the panelists and were hence selected for subsequent evaluations: yoghurt-like, fatty, buttermilk-like, cream-like, sour cream-like and cream cheese-like. At Week 0, the odor attribute “yoghurt-like” was described for all samples as being clearly to strongly perceivable. All the other odor attributes were perceived rather weakly in the yoghurts, with just a few exceptions: the attributes, fatty, cream-like and cream cheese-like, were described for the RF 58 °C yoghurt as being slightly more strongly perceivable than in the other yoghurts. The attributes, buttermilk-like and sour cream-like, were evaluated highest in the case of the set-style reference yoghurt. A closer look at Table 4 reveals that only three attributes were evaluated as being significantly different between the products (Week 0): yoghurt-like, cream-like and cream cheese-like. In detail, those variances arose either between the references and the heat-treated samples (yoghurt-like, cream-like) or between the yoghurts heated to 58 °C and the other products (cream cheese-like). After two and four weeks of storage, these differences decreased and were no longer significant. Hence, during storage, the aroma profiles of the reference and heated yoghurts were found to be very similar to each other.

**Table 4:** Retronasal intensity rating of specific flavor attributes by aroma profile analysis (APA) of yoghurt gels with/without additional heat treatment after culturing and the results of ANOVA (one-way): Weeks 0, 2 and 4.

Aroma profile analysis (APA) (scale: 0–3; mean values)		Reference yoghurt	RF-treated yoghurts		CV-treated yoghurts		Set reference	ANOVA results	
			58 °C	65 °C	58 °C	65 °C		*F* value	*p* value
Week 0	yoghurt-like	2.6 a	1.6 c	2.0 b,c	2.1 b	2.2 a,b	2.3 a,b	4.27	1.86 × 10^−3^
	fatty	1.3	1.6	1.2	1.1	1.1	0.9	1.22	3.10 × 10^−1^ *
	buttermilk-like	0.9	1.2	1.3	0.9	1.2	1.4	0.78	5.70 × 10^−1^ *
	cream-like	0.6 c	1.3 a	0.7 b,c	1.2 a,b	0.7 b,c	0.3 c	3.53	6.53 × 10^−3^
	sour cream-like	0.6	1.1	1.1	0.8	1.1	1.4	1.28	2.80 × 10^−1^ *
	cream cheese-like	0.6 b	1.4 a	0.6 b	1.0 a,b	0.8 b	0.8 b	2.60	3.24 × 10^−2^
Week 2	yoghurt-like	2.3	1.8	2.1	2.0	1.7	-	1.01	4.12 × 10^−1^ *
	fatty	1.2	0.9	1.1	0.7	0.9	-	0.74	5.71 × 10^−1^ *
	buttermilk-like	0.9	0.8	1.0	1.0	1.3	-	0.51	7.26 × 10^−1^ *
	cream-like	0.8	0.9	1.1	0.9	1.0	-	0.29	8.82 × 10^−1^ *
	sour cream-like	1.3	1.0	1.0	1.1	1.1	-	0.40	8.11 × 10^−1^ *
	cream cheese-like	0.7	1.2	1.0	1.3	1.3	-	1.08	3.73 × 10^−1^ *
Week 4	yoghurt-like	2.2	2.0	2.2	2.3	2.3	-	0.27	8.98 × 10^−1^ *
	fatty	1.0	0.8	0.7	1.0	0.8	-	0.13	9.70 × 10^−1^ *
	buttermilk-like	1.1	0.8	1.5	1.1	1.1	-	0.87	4.90 × 10^−1^ *
	cream-like	0.5	0.6	0.5	0.4	0.3	-	0.74	5.68 × 10^−1^ *
	sour cream-like	0.9	1.0	1.0	0.8	1.2	-	0.36	8.34 × 10^−1^ *
	cream cheese-like	0.7	1.0	0.9	1.2	1.1	-	0.59	6.72 × 10^−1^ *

* No post-hoc test necessary (*p* ≥ 0.10, n.s.). Intensity values with different letters indicate significant differences between products (*p* < 0.05, Fisher LSD post-hoc).

A taste profile analysis was additionally performed with the results being shown in Table 5. At Week 0, all yoghurt products were described as being sour with medium intensity (4.5–6.3); thereby, the set-style reference was evaluated as being the sourest (6.3). The attributes sweet, salty and bitter were just slightly perceivable and, in general, no significant differences between the samples could be detected. Thus, no formation of bitter peptides in the course of the additional heating step was observed. Additionally, no significant differences between the products became evident. In storage, the taste profiles of the samples remained quite close to each other, and no significant differences arose. Accordingly, it can be concluded that the trained sensory panel not only evaluated the aroma, but also the taste profiles of all heat-treated samples as being very close to the stirred reference product.

**Table 5:** The intensity rating of specific taste attributes by taste profile analysis of yoghurt gels with/without additional heat treatment after culturing and the results of ANOVA (one-way): Weeks 0, 2 and 4.

Taste profile analysis (visual analogue scale, mean values)		Reference yoghurt	RF-treated yoghurts		CV-treated yoghurts		Set reference	ANOVA results	
			58 °C	65 °C	58 °C	65 °C		*F* value	*p* value
Week 0	sweet	1.8	2.0	2.2	2.3	2.1	1.3	0.55	7.37 × 10^−1^ *
	sour	5.3	5.6	4.9	4.5	5.2	6.3	1.07	3.85 × 10^−1^ *
	salty	1.2	0.8	1.0	1.0	0.9	1.1	0.09	9.93 × 10^−1^ *
	bitter	0.7	0.9	1.0	0.5	0.5	0.9	0.34	8.84 × 10^−1^ *
Week 2	sweet	1.5	0.9	1.8	1.9	1.2	-	0.76	5.55 × 10^−1^ *
	sour	5.9	5.8	5.8	5.3	6.5	-	0.47	7.56 × 10^−1^ *
	salty	1.2	1.4	1.3	1.4	1.5	-	0.04	9.97 × 10^−1^ *
	bitter	0.4	0.2	0.2	0.2	0.2	-	0.26	9.05 × 10^−1^ *
Week 4	sweet	0.9	1.7	2.0	1.3	1.2	-	1.15	3.45 × 10^−1^ *
	sour	5.1	5.6	5.5	5.2	5.4	-	0.12	9.76 × 10^−1^ *
	salty	0.9	1.1	0.9	1.5	0.7	-	0.51	7.29 × 10^−1^ *
	bitter	0.5	0.5	0.6	0.5	0.7	-	0.12	9.74 × 10^−1^ *

* No post-hoc test necessary (*p* ≥ 0.10, n.s.). Intensity values with different letters indicate significant differences between products (*p* < 0.05, Fisher LSD post-hoc).

Moreover, with the storage of the individual yoghurts, no significant changes in the flavor or taste profiles were detected. The only exceptions were the 58 °C samples (RF, CV) in the case of the attribute cream-like: its intensity was evaluated as significantly higher in the samples at Week 0, but a subsequent decrease occurred thereafter. Generally, the observed lack of changes in flavor (intensity) in storage is in good agreement with previous finding [16,24]. Martin *et al.* [24] reported very low differences in the typical retronasal and orthonasal odor profiles of yoghurt (e.g., yoghurt-like, cream-like, butter-like) during 21 days of storage. Only the attribute, cream-like, was rated significantly lower in storage, and the same was observed for the 58°C heated products in the present study. Overall, Martin *et al.* [24] reported only minor olfactory differences in the aroma profiles of such plain yoghurt products fermented using different starter cultures. In that work, only the odor attribute, yoghurt-like, was ranked with medium intensity, and all other attributes were found to be only weakly perceivable, showing, again, the overall agreement with the findings of the present study.

In the present study, triangular testing was additionally performed to determine whether general differences in the sensory profiles of the differently treated yoghurts were statistically significant at the end of their predicted shelf-life (Week 4). To avoid any influence of the yoghurts appearance on the results of the triangular evaluation (e.g., due to slight color differences, as reported in Section 3.4), the testing was performed blindfolded by the panelists. In detail, two triads were tested: reference *vs*. RF 65 °C and RF 58 °C *vs*. CV 58 °C. The first triad was chosen to compare the reference yoghurt with an RF heated yoghurt, since this treatment was generally found to be very mild in the case of 58 and 65 °C (Section 3.3). The second triad was chosen to reveal the differences between RF and CV heated products for the mildest temperature of 58 °C, because huge varieties in heating rates were found (Section 3.1 and Section 3.2). In accordance with the results of the APA and the taste profile analysis, the tests confirmed that all of the samples were very close to each other with respect to their overall sensory properties: for none of the triads was the divergent sample identified with statistical significance. Out of 24 evaluations, 13 correct answers would have been necessary to indicate a significant difference between the samples at a level of significance of 5% (alpha and beta error, respectively). In the case of the triad, reference *vs*. RF 65 °C, 12 out of 24 evaluations led to the correct identification of the divergent sample; for the triad, RF 58 °C *vs*. CV 58 °C, only seven evaluations identified the divergent sample correctly. Accordingly, the samples were not distinguishable based on their overall sensory properties.

## 4. Conclusions

The present study evaluated the effect of additional heat treatment (CV and RF) after the fermentation process in yoghurt production. Such additional heat treatment has been little studied previously. Both heating methods showed promise concerning the homogeneity of the temperature distribution. However, RF appeared to be superior, due to a very fast heating rate of 0.3 K·s^−1^. This study also focused on the yoghurt shelf-life, pH, color and sensorial quality. Microbial characterization demonstrated the mildness of the RF treatment, because LAB partially survived. Nevertheless, a reduction in LAB highlighted the potential of this method for prolonging the yoghurt shelf-life, not only by inactivating the yeasts and molds, but also by preventing strong post-acidification. Whereas generally, no significant changes in pH and sensorial quality were observed, slight color changes were found to occur after heat treatment, possibly due to the onset of the caramelization processes. Based on these observations, we believe RF heating is a promising technological tool for future applications in the dairy industry (e.g., milk pasteurization, sterilization or post-heating of fermented products). However, further improvements with regard to batch-wise manufacturing are necessary in order to open up this technique to industry, such as the application of radio frequencies in a continuous process. 

## Abbreviations

ANOVA: analysis of variance
APA: aroma profile analysis
CFU: colony forming units
CV: convectional heating
ε_r_: relative permittivity/relative dielectric constant
LAB: lactic acid bacteria
n.s.: not significant
RF: radio frequency heating
SD: standard deviation
sig.: significant
